# CRISPR base editor treats premature-aging syndrome

**DOI:** 10.1038/s41392-021-00576-6

**Published:** 2021-04-16

**Authors:** Ping Lin, Jianxin Jiang, Min Wu

**Affiliations:** 1 Wound Trauma Medical Center, State Key Laboratory of Trauma, Burns and Combined Injury, Daping Hospital, Army Medical University, Chongqing, China; 2 Department of Biomedical Sciences, School of Medicine and Health Sciences, University of North Dakota, Grand Forks, ND USA

**Keywords:** Genetic engineering, Genetic techniques, Gene therapy

**Fig. 1:**
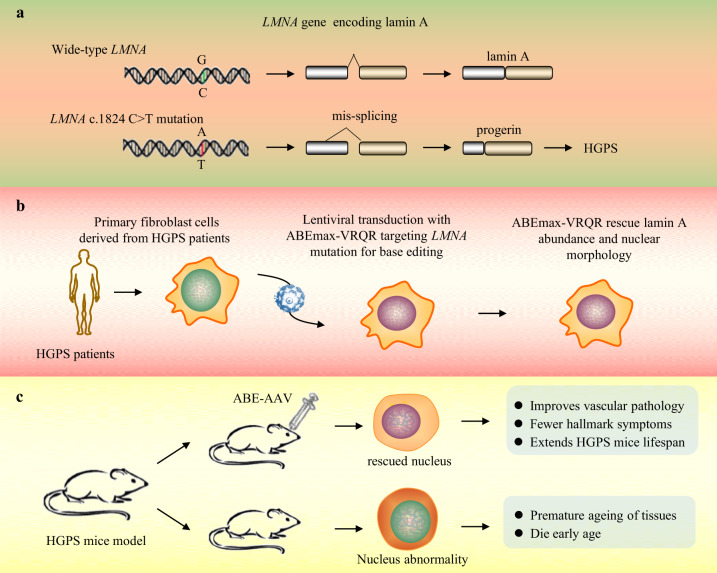
Programmable base editing rescues Hutchinson–Gilford progeria syndrome. **a** A single base mutation at 1824 locus in *LMNA* gene results in mis-splicing and subsequently translates into a truncated lamin A protein, termed progerin, which causes Hutchinson–Gilford progeria syndrome (HGPS). **b** ABEmax-VRQR corrected human *LMNA* mutation in two primary fibroblast cells derived from HGPS patients. Using the lentiviral delivery delivered ABEmax-VRQR and sgRNA targeting *LMNA* c.1824 C > T mutation, resulting in gene editing of the *LMNA* loci and subsequently rescuing lamin A abundance and nuclear morphology. **c** ABEmax-VRQR treatment in HGPS mice model. Koblan et al. used the clinical adeno-associated virus vector for co-packaged ABEmax-VRQR to make the correction of mutation in many tissues, but not all, in mouse model, resulting in improved vascular pathology and extended the HGPS mice lifespan

A recent paper published in *Nature* by Koblan et al. reported the use of CRISPR-mediated adenine base editor (ABE) to repair mutations of the Hutchinson–Gilford progeria syndrome (HGPS or progeria), attenuate symptoms, and extend lifespan of mice (Fig. 1),^1^ representing a major advance in design of treatments for human accelerated-ageing disorders and potentially other genetic diseases.

HGPS is an extremely rare but incurable genetic disease exhibiting rapid aging of multiple organs, ultimately leading to early death (patients die at teens or early 20s). Most HGPS patients exhibit a single base mutation in the lamin A (LMNA) gene from cytosine (C) to a thymine (T) (1824C > T) (Fig. 1a). This mutation leads to mis-splicing during *LMNA* transcription and subsequently translates into a truncated lamin A protein, termed progerin (Fig. 1a), in which progerin protein always keeps tagging with a farnesyl group. The accumulation of farnesylated progerin is toxic for nuclear shape and rigidity, which hampers nucleus function resulting in HGPS disease. Attempts to discover treatments for progeria initially focused on trying to reduce the accumulation of farnesylated progerin. Although small-molecule inhibitors for farnesyltransferase have been screened and tested to provide treatment options for patients, the treatment with them only partially alleviates the disease symptoms in clinics. Beyret et al. and Santiago-Fernández et al. have used CRISPR-Cas9-mediated lamin A/progerin reduction by disrupting activity of HGPS-mutated gene. But their health reverted only several alterations in HGPS cells and mice. To make the matter worse, these treatment strategies also lead to frameshift mutations in the *LMNA* gene, which could disable a human’s copy of the gene to adequately correct the disease due to this harmful effect. Therefore, it is necessary to develop a new strategy to directly repair the mutation that causes HGPS.

CRISPR-Cas biotechnology, gene-modifying, and engineering are revolutionizing the ways of treating genetic diseases by correcting genetic mutations, which facilitate gene editing to treat or cure certain inheritable diseases, cancers, and other illnesses. ABE, using a catalytically impaired Cas9 fused with evolved deoxyadenosine deaminase, mediates the conversion of targeted A•T to G•C through an intermediated that reads as guanine (G) by polymerases during DNA replication.^2^ Koblan et al. used an optimized ABE7.10 variant (ABEmax-VRQR)^3^ with sgRNA targeting c.1824 C > T in *LMNA* (Fig. 1b).^1^ Through the lentiviral delivery, Koblan et al. tested the ABEmax-VRQR in two primary cells derived from HGPS patients, resulting in the correction of ~90% of the mutated genes.^1^ This led to normal splicing of laminA, reduced a notable expression and accumulation of progerin, and substantially corrected the abnormal nucleus (Fig. 1b).^1^ Thus, the ABE approach developed in this study could repair the mutated gene in HGPS cells.

Next, to investigate the use of ABE treatment to correct the HGPS mutation in vivo, Koblan et al. used the clinical adeno-associated virus vector for co-packaged ABEmax-VRQR and sgRNA (ABE-AAV) to deliver the repair complex machinery into the HGPS mouse model (Fig. 1c)^1^—C57BL/6 mice homozygous includes the pathogenic human *LMNA* c.1824 C > T allele that develops many hallmark symptoms seen in human HGPS patients, such as loss of vascular smooth muscle cells (VSMCs), cardiovascular complication, loss of subcutaneous fat and early death.^4^ Following the retro-orbital injection of ABE-AAV in mice at postnatal day 3 (P3) and 14 (P14), Koblan et al. achieved a 10–60% correction of the human *LMN*A mutation gene in numerous organs at 6 weeks of age (Fig. 1c).^1^ Remarkably, higher editing efficiency was observed in bone, skeletal muscle, live, heart, and aorta when the mice were examined 6 months later.^1^ In addition, the base editing approach resulted in a reduction in progerin transcript abundance and an increase in lamin A levels in the liver, heart, and aorta tissues.^1^ Importantly, compared to HGPS mice with severe vascular disease, the HGPS mice treated with ABE-AAV showed greatly improved aortic health without fibrous tissue growth, restoring adventitial thickness and counts of VISMCs (Fig. 1c).^1^ Furthermore, ABE-AAV treatment also showed a greatly extended lifespan of HGPS mice (Fig. 1c).^1^ Together, ABE treatment attenuates some typical symptoms of HGPS disease in vivo, which may extend the life expectancy in the diseased mice.

Correcting the mutation in HGPS mouse models provides a potential utility of ABE as a possible therapeutic approach to cure human HGPS. However, are ABE-treated cells safe in humans? One of the safety concerns is the control of undesired genome editing outcomes, because excessive, prolonged expression of an active ABE system could increase the off-target effect. Cas-independent off-target DNA editing by ABE7.10 has been reported to be minimal or undetectable.^2^ To assess off-target editing by ABEmax-VRQR, Koblan et al. performed CIRCLE-seq on genomic DNA and next-generation RNA sequencing (RNA-seq) on RNA isolated from ABE-lentivirus-treated HGPS patient cells, suggesting a minimal degree of off-target DNA or RNA editing and undetectable for the top 32 candidate off-target loci for the selected sgRNA.^1^

An important question is that therapeutic editing requires effective and targeted delivery methods in human HGPS patients. Koblan et al. used the AAV, a delivery modality that is approved by the US Food and Drug Administration (FDA), to express the base editor.^1^ The authors chose the AAV9 capsid for its broad tissue tropism, clinical validation, and ability to transduce into progeria-inflicted cells. However, AAV gene therapy may have the potential for genotoxic integration events. A long-term study of AAV8 or AAV9 gene therapy reveals that 1741 unique integration events were observed in genomic DNA, which clonal expansion of cells is associated with cancer in humans.^5^ Therefore, it is necessary to further assess the safety of AAV gene therapy in humans. To our knowledge, both lipid nanoparticles (LNP) and electroporation (EP) delivery methods encapsulate modified mRNA or DNA molecules of ABEmax-VRQR and its sgRNAs could be as alternative strategies instead of the AAV delivery method due to the recent success in the fastest development of mRNA vaccines for COVID-19 using LNP in our lifetime. In addition, the delivery efficiency and specificity of gene editing for the pathogenic human *LMNA* c.1824 C > T allele need to be tested in clinical application, which is challenging and often involves years of try and error practice. Moreover, what is the optimal distribution of ABEmax-VRQR, and which organs can be targeted by distinct delivery methods (AAV, LNP, EP or others)? Although in vivo delivery for therapies remains many challenges, we anticipate that further testing could determine the suitable delivery for gene therapy to control diseases and improve human health.

Another safety concern is that whether ABEmax-VRQR, a bacterial protein, will stimulate immune response. Cas9-induced immune responses have been reported in general human populations. Therefore, a response may lead to non-active treatment if cells containing the components of ABEmax-VRQR, such as Cas9, were selectively eliminated. However, a long-term clinical study showed that the minimal levels of Cas9 (<0.75 fg per cell) did not activate strong Cas9-specific humoral immunity, providing a hope for the treatment dose of ABEmax-VRQR for no immunogenic administration in clinical applications. Furthermore, using immune-orthogonal orthologues of Cas9, discovering new CRISPR-Cas enzymes, or engineering Cas9 protein with mapping and editing epitopes for a better immunological profile, might circumvent immune response in humans. In addition, AAV-mediated CRISPR therapeutics also face the same safety concern, such as existing humoral and cellular immunity against AAV capsid. Therefore, it remains to be investigated whether delivering ABEmax-VRQR into the human body will compromise safety or therapeutic efficacy. Overall, focusing on the clinically validated long-term safety of ABE-AAV and its-edited cells in humans will contribute to the way for next-generation gene therapies.

The ethical concerns for CRISPR-Cas9 genome editing technology, including the future of the modified organisms, moral decisions, etc., have long been discussed by normal citizens, scientists, and ethicists. If ABE is to be used to cure human disease, whether and how legalize experimentation on human somatic and germline cells? What guidelines from national and international organizations should be crafted to govern its application in humans? And what extent is ABE allowed to be used in translational and clinical medicine? Going forward, establishing an organization to decide how best to address these ethical complexities of ABE application and other gene-editing approaches for the betterment of human health and progress is urgent.

Taken together, the current preliminary results from Koblan et al. demonstrate that ABE treatment has potential to correct the HGPS mutation in mice, fixing phenotypic traits and extending animal lifespan in a mouse model. These findings suggest a potential future functional base editing treatment for human HGPS and possibly other genetic disorders.
